# Severe Acute Respiratory Syndrome Coronavirus 2 (SARS-CoV-2)-Induced Cardiovascular Syndrome: Etiology, Outcomes, and Management

**DOI:** 10.7759/cureus.8543

**Published:** 2020-06-10

**Authors:** Alla Turshudzhyan

**Affiliations:** 1 Internal Medicine, University of Connecticut Health Center, Farmington, USA

**Keywords:** cardiomyopathy, covid-19, sars-cov-2, takotsubo

## Abstract

As the coronavirus disease 2019 (COVID-19) pandemic evolves, more complications associated with the disease come to surface. Thus far, there is limited information available on the etiology, clinical outcomes, and management options for cardiovascular complications caused by COVID-19. This review focuses on literature published in year 2020 on the virus-induced cardiovascular damage with intention to better understand pathophysiology of this process, its impact on clinical outcomes, and available therapies. Literature review shows that severe acute respiratory syndrome coronavirus-2 (SARS-CoV-2) acts through angiotensin-converting enzyme 2 (ACE-2) receptors and causes cardiac injury by direct damage to the cardiomyocytes, systemic inflammation, fibrosis, interferon and cytokine-mediated immune response, coronary plaque destabilization, and hypoxia. Comorbidities, especially underling heart disease, make patients more predisposed to severe cardiovascular damage. COVID-19 patients who develop myocardial injury have a higher mortality rate compared to those who do not. During the pandemic, percutaneous coronary intervention (PCI) should remain the standard of care for patients with ST segment elevation myocardial infarction (STEMI). On the other hand, in order to limit healthcare worker exposure, patients with non-ST segment elevation myocardial infarction (NSTEMI) should be managed with stabilization strategies if hemodynamically stable. Monitoring hospitalized COVID-19 patients with high sensitivity troponin can help screen for severe complications and detect them early. Use of multiple investigational drugs with uncertain cardiac safety profiles in COVID-19 patients requires continuous cardiac monitoring. Notch signaling pathway therapy along with anti-viral agents, interleukin-6 inhibitors, and convalescent serum are possible treatment options to better control the inflammatory state that drives the cardiac damage.

## Introduction and background

As the number of coronavirus disease 2019 (COVID-19) cases grows, more complications associated with the disease become apparent. One of the more concerning complications affects the cardiovascular system. Thus far, there is limited information available on the etiology, clinical outcomes, and management options for cardiovascular complications caused by COVID-19. The more common cardiovascular sequalae are acute coronary syndrome, cardiomyopathy, arrythmia, myocarditis, cardiogenic shock, and cardiac arrest [1]. Interestingly, the observed cardiovascular injury is similar to that caused by Middle East respiratory syndrome coronavirus (MERS-CoV), severe acute respiratory syndrome coronavirus 1 (SARS-CoV-1), and influenza [1]. This review focuses on literature published in the year 2020 on COVID-19 induced cardiovascular complications with intention to better understand pathophysiology of this process, its impact on clinical outcomes, and available therapies. 

## Review

In order to understand management options, clinical outcomes, and mortality associated with COVID-19 induced cardiovascular injury, it is important to first better understand the pathophysiology. Babapoor-Farrokhran et al. highlighted resemblance in pathophysiology of severe acute respiratory syndrome coronavirus 2 (SARS-CoV-2) with SARS-CoV-1 and MERS-CoV [2]. They concluded that in all three coronavirus outbreaks, angiotensin-converting enzyme 2 (ACE-2) receptors played an important role. Disruption of these receptors causes cardiomyopathy and leads to heart failure. Not only has COVID-19 been linked to the development of severe cardiovascular complications, but it was also found to have affinity for patients with underlying cardiovascular disease, who are at risk for developing severe complications. The mechanism of myocardial injury caused by COVID-19 has not been fully understood, but the proposed mechanism is thought to be through direct damage to cardiomyocytes, systemic inflammation, fibrosis, interferon and cytokine-mediated immune response, coronary plaque destabilization, and hypoxia [2].

Rizzo et al. also emphasized the relationship between COVID-19 and development of cardiac disease. In their work, they further described the pathophysiology of cardiovascular injury in COVID-19 patients through notch signaling that is implicated in both immune response to the viral infection and regulation of cardiovascular function. This is an important target for therapies and is discussed later in this review. Etiology for cardiovascular complications in COVID-19 patients suggested by Rizzo et al. is in line with what Babapoor-Farrokhran et al. proposed earlier, but they also commented that cardiac injury could be due to microvascular damage and stress-related cardiomyopathy. Rizzo et al. further explained that SARS-CoV-2 has potential to directly replicate within cardiomyocytes and pericytes causing viral myocarditis [3].

Hendren et al. proposed that in patients with no underlying coronary artery disease cardiomyopathy, ventricular arrythmias, and hemodynamic instability are the more common presentations of cardiovascular complications [4].

A number of case series and case studies have been done to help assess risk factors, outcomes, and mortality related to COVID-19 induced cardiovascular complications. A case series of 21 patients from Washington state with a mean age of 70 years and 52% male was presented by Arentz et al. They concluded that comorbidities were identified in 86% of cases, most commonly chronic kidney disease and congestive heart failure, with cardiomyopathy developing in 33% of cases [5]. Shi et al. evaluated 416 patients with COVID-19, of whom 19.7% had evidence of myocardial injury. Patients with myocardial injury had a significantly higher mortality rate at 51.2% when compared to patients without myocardial injury at 4.5%. In patients with myocardial damage, a greater degree of elevation in high-sensitivity troponin I levels was associated with higher mortality rates [6]. Guo et al. researched the impact of pre-existing cardiovascular disease (CVD) and new myocardial injury on fatal outcomes in COVID-19 patient population. Of the 187 patients in retrospective single-center case series they evaluated, 27.8% developed a new myocardial injury causing cardiac dysfunction and arrhythmias. They concluded that while myocardial injury is associated with fatal outcomes in COVID-19 patients, clinical prognosis for patients with pre-existing CVD but without superimposed myocardial injury is favorable. Based on these results, COVID-19 patients with known underlying CVD and evidence of myocardial injury require more aggressive therapies [7]. 

Interestingly, there have been a few cases of Takotsubo cardiomyopathy, also known as broken heart syndrome, in COVID-19 patients. Takotsubo cardiomyopathy oftentimes presents similar to an acute myocardial infarction with a marked elevation in troponins and motion wall abnormalities on echocardiogram but no coronary artery disease on catheterization. Chadha reported a case of Takotsubo in an 85-year-old female with no underlying cardiac or pulmonary disease. The reported case was brought on by severe anxiety over the current COVID-19 pandemic [8]. Dabbagh et al. presented another case of Takotsubo cardiomyopathy from COVID-19 that was further complicated by large hemorrhagic pericardial effusion with echocardiographic signs of tamponade. This patient responded well to treatment with pericardiocentesis, steroids, hydroxychloroquine, and colchicine [9]. Minhas et al. and Kir et al. reported observed cases of Takotsubo in COVID-19 patients, with the case presented in Kir et al. also manifesting in rarely seen high-grade atrioventricular block [10,11].

Given high mortality and poor outcomes associated with cardiovascular complications from COVID-19, available treatment options need to be discussed. It is important to have a management plan for ST segment elevation myocardial infarction (STEMI) and non-ST segment elevation myocardial infarction (NSTEMI) during the pandemic. Mahmud et al. emphasized the importance of keeping COVID-19 induced cardiac complications in mind when evaluating patients with ST elevations and choosing reperfusion therapy. During the pandemic, percutaneous coronary intervention (PCI) should remain the standard of care for patients with STEMI, and if PCI is not available, fibrinolysis should be considered. On the other hand, in order to limit healthcare worker exposure, patients with NSTEMI should be managed with stabilization strategies if hemodynamically stable. Given acuity of these interventions and oftentimes lack of known COVID-19 status, it is important that all healthcare professionals are adequately attired with personal protective equipment if the patient has STEMI and PCI is necessary [12]. Hendren et al. also recommended a conservative approach with no extensive cardiac workup for COVID-19 patients with elevated troponin but who are otherwise medically stable. On the other hand, they summarized that COVID-19 patients with ventricular arrythmias and hemodynamic instability require a full cardiological evaluation and consideration for experimental therapies and clinical trials [4].

While cardiovascular sequelae bear poor outcomes and high mortality for COVID-19 patients, there are no guidelines on early screening or prevention of these complications. Atallah et al. recommended screening COVID-19 patients using high-sensitivity troponin to better triage patients into high- or low-risk groups. If symptomatic, patients should be monitored with troponin daily and if troponin is normal, levels can be monitored longitudinally to predict potentially worsening disease. Because surviving patients had minimal elevation in troponin levels, using a high-sensitivity troponin to screen for possible severe complications and poor prognosis early may be beneficial [13].

Naksuk et al. expressed concern that in addition to virus-induced cardiovascular damage, therapy that is currently being used for treatment of COVID-19, such as chloroquine and hydroxychloroquine, can cause irreversible cardiomyopathy or worsen underlying cardiac dysfunction. Both chloroquine and hydroxychloroquine along with other therapies used, such as favipiravir, lopinavir/ritonavir, macrolide agents, and piperacillin-tazobactam, can cause atrioventricular block, bundle branch block, and QTc prolongation. Due to multiple investigational and off-label drug use with uncertain cardiac safety profiles and limited resources for cardiac monitoring and drug-drug interaction, there should be clear protocols of how these therapies should be used and cardiac monitoring should be provided to patients on multiple agents that could affect the heart function [14].

An important therapy target was previously discussed by Rizzo et al. Notch signaling pathway is implicated in both immune response to virus infection and regulation of cardiovascular function. SARS-CoV-2 entry into the cell is facilitated by binding of the viral spike glycoprotein to the ACE-2 receptor, followed by the proteolytic cut of the S glycoprotein by the host protease furin. The number of ACE-2 receptors on cell membrane is regulated by ADAM17. The Notch signaling is a positive regulator of furin and negative regulator of ADAM17. Prevention of Notch signaling pathway activation can interfere with viral entry into the cells by reducing furin and increasing ADAM17. Notch signaling pathway can be used as a possible target therapy to prevent SARS-CoV-2 infection from progressing to cardiac disease [3]. Hendren et al. proposed that prior experience with SARS-CoV-1 may guide current management of cardiovascular complications with anti-viral agents, interleukin-6 inhibitors, and convalescent serum [4].

## Conclusions

Cardiovascular complications from COVID-19 are quickly becoming one of the more concerning sequelae of this disease. Just like the previous outbreaks of coronavirus, SARS-CoV-2 acts through ACE-2 receptors and causes cardiac injury though direct damage to the cardiomyocytes, systemic inflammation, fibrosis, interferon and cytokine-mediated immune response, coronary plaque destabilization, and hypoxia. Comorbidities, especially underling heart disease, make patients more predisposed to severe cardiovascular complications. COVID-19 patients who develop myocardial injury have a higher mortality rate compared to those who do not. Interestingly, a number of Takotsubo cardiomyopathy cases related to COVID-19 have been reported. More research needs to be done to further assess that association. It is important to consider COVID-19 induced cardiac injury when evaluating STEMI and NSTEMI. For STEMI, PCI should remain the standard of care, while NSTEMI, given hemodynamic stability, should be managed with stabilization strategies in order to limit healthcare worker exposure. Monitoring hospitalized COVID-19 patients with high sensitivity troponin can help screen for possible severe complications early. Multiple investigational and off-label drugs with uncertain cardiac safety profiles are currently being used in the treatment of COVID-19; therefore, telemetry should be provided to patients on multiple agents. Notch signaling pathway is an important target for therapy to prevent cardiovascular complications. Other therapies that can be used to control the cardiovascular injury from COVID-19 are anti-viral agents, interleukin-6 inhibitors, and convalescent serum. More research needs to be done to assess effectiveness of these therapies.
